# A Qualitative Study Exploring Perceptions to the Human T Cell Leukaemia Virus Type 1 in Central Australia: Barriers to Preventing Transmission in a Remote Aboriginal Population

**DOI:** 10.3389/fmed.2022.845594

**Published:** 2022-04-29

**Authors:** Fiona Fowler, Lloyd Einsiedel

**Affiliations:** ^1^Department of Social Work, Alice Salomon University of Applied Sciences, Berlin, Germany; ^2^Alice Springs Hospital, Alice Springs, NT, Australia

**Keywords:** HTLV-1, infectious diseases, Central Australia, Indigenous health, decolonizing

## Abstract

**Background:**

Central Australia has the highest recorded prevalence of infection with the human T cell leukaemia virus type 1 (HTLV-1) worldwide. Each of the clinical diseases associated with HTLV-1 have been reported in this region, including deaths due to adult T cell leukaemia, which is causally linked to HTLV-1. Nevertheless, no public health response has been implemented to reduce HTLV-1 transmission among the affected Aboriginal population. In the first study to explore the perceptions of healthcare professionals along with those of Aboriginal people whose communities are actually impacted by HTLV-1, we sought to understand the barriers to preventing HTLV-1 transmission in this remote area.

**Methodology/Principal Findings:**

Semi and un-structured interviews were conducted with 30 Australian Aboriginal people, 26 non-Aboriginal healthcare professionals and 3 non-Aboriginal community workers. The purpose of the interviews was to explore perceptions towards HTLV-1 in a health context with a focus on sexual and reproductive rights. Deductive and inductive analyses were applied to the data and a decolonizing lens brought peripheral stories to the fore. A major finding was the contrast between views expressed by Aboriginal participants and healthcare professionals regarding the provision of knowledge to those affected. Aboriginal participants consistently articulated that they and their communities should be informed of, and can hold, knowledges pertaining to HTLV-1. This finding controverted the perceptions of healthcare professionals that the complexities of the virus would not be well-understood by their Aboriginal patients and that sharing HTLV-1 knowledges might overwhelm Aboriginal people. Further analyses revealed a spectrum of understanding and clinical practice, while also delineating signs of an imagined public health response.

**Conclusions/Significance:**

HTLV-1 remains a neglected infection in Australia. Knowledge of HTLV-1 is held by a privileged medical elite and does not flow to marginalised Aboriginal people living in affected communities. We demonstrate that differences in the perspectives of stakeholders presents a significant barrier to the development of cohesive, culturally safe prevention programs that foster a shared knowledge of HTLV-1. The interview data suggests that a successful public health program is likely to require a dual approach that includes clinical care and community-driven health promotion. Aspects of this approach, which would raise awareness and potentially reduce transmission and lower HTLV-1 prevalence in Central Australia, may be applicable to other endemic settings with similar conditions of social disadvantage, geographic remoteness, resource limitations and cross-cultural challenges.

## Introduction

The human T cell leukaemia virus type 1 (HTLV-1) is a human retrovirus that predominantly infects CD4+T cells, which are crucial to the body’s adaptive immune response. HTLV-1 is epidemiologically linked as the cause of two serious diseases; an aggressive haematological malignancy (adult T cell leukaemia/lymphoma, ATL) and a neurodegenerative disease of the spinal cord (HTLV-1 associated myelopathy, HAM) (1, 2). The estimated lifetime risk of developing an HTLV-1 associated disease is generally given as 5%, which is the combined risk of developing ATL and HAM, the two conditions for which data are available [5% and 1–4% for ATL and HAM, respectively (1–5)]. However, this is undoubtedly an underestimate.

Other clinically recognised conditions that contribute to the HTLV-1 disease burden include uveitis, HTLV-1 associated pulmonary disease (HAPD) and infective dermatitis (6–8). Moreover, HTLV-1 seropositivity is associated with an increase in mortality that cannot be attributed to ATL or HAM in all endemic areas where this has been studied (9). In Central Australia, a high baseline number of HTLV-1 copies in peripheral blood leucocytes (proviral load, PVL) prospectively increased the risk of death three to fourfold in hospital based studies, with most of these deaths resulting from complications of chronic pulmonary disease (10, 11).

Infection with HTLV-1 was first reported in an Aboriginal Australian population in 1988 and high infection rates were confirmed in Central Australia in 1992 (12, 13). In this region, each of the recognised clinical entities associated with HTLV-1 have been recorded, including fatalities resulting from ATL (7, 14–19). The average adult community-based prevalence of HTLV-1 among Aboriginal people in Central Australia was recently recorded to be 39% (19); the highest prevalence reported worldwide. In contrast, the overall prevalence of HTLV-1 among blood donors in Australia in the years 2009–2018 was 0.003%, demonstrating that elsewhere in the country HTLV-1 is not endemic (20). Notwithstanding the high infection rates and reports of HTLV-1 associated diseases in Central Australia (14), no systematic public health response has been implemented to reduce transmission among Aboriginal Australian people. Indeed, the perceived value of such an initiative remains controversial among Australian public health professionals (21).

Globally, most infections are thought to result from sexual transmission (22, 23). However, HTLV-1 is also transmitted through blood contact and from mother to child via prolonged breastfeeding, making infection and progression to associated diseases preventable. In the absence of a vaccine or treatment for HTLV-1, preventative approaches are key to an effective public health response. A dramatic reduction in incidence rates followed the introduction of measures targeting mother-to-child transmission and the testing of blood donors in Japan (24). Few jurisdictions have implemented similar strategies to prevent and reduce HTLV-1 transmission. In Central Australia, antenatal testing for HTLV-1 has been discouraged due to a perception that it will “add to the burden” experienced by Aboriginal women (21).

The correspondent “invisibility” of HTLV-1 has been attributed to its silent transmission, an extended latent period before currently recognised clinical manifestations become apparent, the focus by public health professionals and researchers on the relatively low occurrence of ATL and HAM, and the failure to develop direct acting anti-viral therapies (25). This has led some healthcare professionals to doubt the clinical significance of the virus, which in turn deters them from discussing transmission risks and unfavourable health outcomes with patients and their families (26).

An additional feature that is instrumental in perpetuating the anonymity of this virus is its occurrence in poor communities, which is the case in South America, the Caribbean, Sub-Saharan Africa and areas of Oceania, including Central Australia (27–29). HTLV-1 infection is closely linked to socio-economic disadvantage (30–32) and particularly in Central Australia, with the ongoing impact of colonization. Consequently, the voices of those affected are seldom heard and the lived experience of HTLV-1 is interpreted by a privileged medical profession that is largely drawn from a very different social context (33). Acknowledging the power of the latter group in influencing perceptions toward HTLV-1, engaging clinicians and public health professionals in HTLV-1 education has been highlighted as the essential first step towards changing the lives of people living with HTLV-1 (34).

Qualitative research, which is derived from the study of the human experience, is recognised for its value in illuminating and addressing important socio-systemic dilemmas. In health, including Aboriginal health, qualitative research cultivates socially and culturally informed hypotheses for larger clinical studies, guides the development of public health interventions through the establishment of grounded theory, and engages patients as partners (35–37). Qualitative research holds value for the development of HTLV-1 knowledges that are context-specific, and patient-centred. The first qualitative study in HTLV-1 explored knowledges and awareness of HTLV-1 among breastfeeding Noir-Marron women in French Guiana (38). More recently, research groups in Brazil have utilised oral history techniques to examine the experiences of HTLV-1 patients, critiquing the biomedical focus on disease risk at the expense of prevention and family support (26), interrogating the failure of healthcare providers to support mothers with decisions about breastfeeding (39), exploring how HTLV-1 diagnosis and illness impacts on the sexual relationships of affected women (40) and influences the reproductive decisions of prospective parents (41), and highlighting the adverse impacts of HAM on social and emotional wellbeing (42). Similar studies have not been done in Australia and the perceptions of clinicians who control the HTLV-1 narrative have not been explored in any context to date.

Our study adds to this small body of research a descriptive account of HTLV-1 perceptions as shared by healthcare professionals and an Australian Aboriginal community cohort in Central Australia. We chose qualitative methods for their strength in providing an avenue for Aboriginal people to make visible their experiences and gain control over a health issue by which they are disproportionately affected. Storytelling methods according to their Aboriginal and Western iterations are the means by which participants were invited to engage in this process, and decolonization, the practice of “undoing” colonialism, was the principle theoretical framework through which the non-Aboriginal researchers sought to understand the themes that emerged (43–47).

The findings of this study justify narrative enquiry as a “two-world” medium through which to explore experiences of heath care in a colonized context (48). While significant barriers to a public health response were identified in the perceptions offered by some healthcare professionals, further participant contributions offered a margin in which to probe opportunities for prevention that are both community-led and clinically informed.

## Materials and Methods

### Study Setting

**FIGURE 1:**
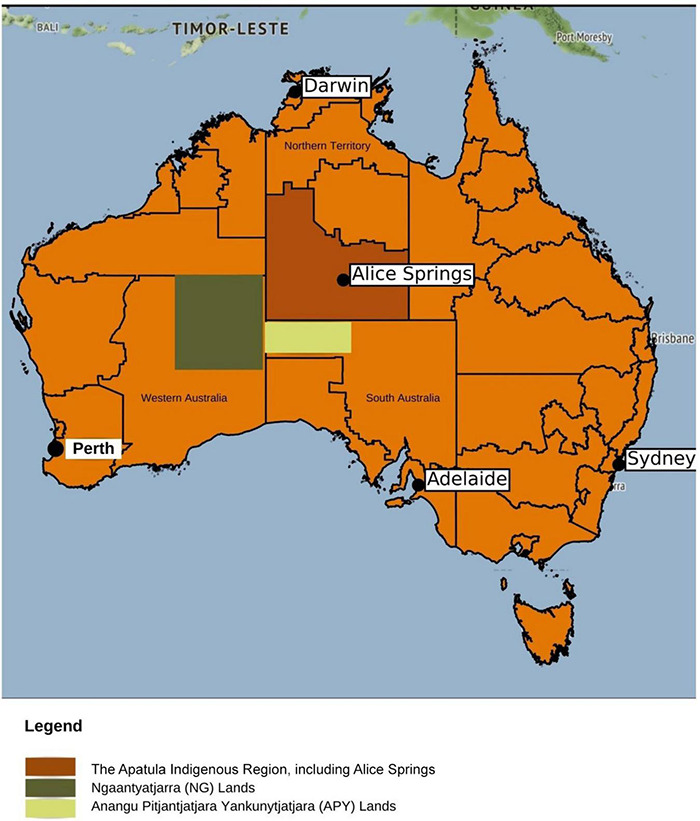
Map of Central Australia. Central Australia covers a remote tri-state region of more than 500 million square kilometres. It includes the vast geographies and diverse language groups of the Ngaanyatjarra (NG) lands, the Anangu Pitjantjatjara Yankunytjatjara (APY) lands and the southern pans of the Northern Territory, including the health hub and regional centre of Alice Springs. These are shown by the shaded sections in above map. *Map not to scale. Modified figure © OpenStreetMap contributors.

Central Australia covers a large, arid landmass of some 500 million km^2^. It is home to more than 500 small, remote Aboriginal communities and some 22 language groups (49, 50) (see Figure 1). Communities are serviced by several primary health care organisations and one community-based hospital in the remote centre of Alice Springs (Alice Springs Hospital, ASH). Thirty-six individual and 5 group interviews were conducted with a total of 59 participants: 30 Aboriginal people and 29 non-Aboriginal healthcare or community workers. Aboriginal participants were recruited from Alice Springs and from a very remote Aboriginal community with an estimated adult resident population (ERP) of 140 (51). All participants had lived or worked in Central Australia (Tables 1, 2).

**TABLE 1:** Profile of Aboriginal participants.

	Sex	Primary language(s)	Location	Previous HTLV-1 knowledges	Interview format	Interview medium
1	F	Pitjantjatjara	Alice Springs		Individual	F2F
2	F	Pitjantjatjara	Alice Springs		Group	F2F
3	F	Warlpiri	Alice Springs		Group	F2F
4	F	Arrernte, Luritja	Alice Springs	Y	Group	F2F
5	F	Arrernte	Alice Springs		Group	F2F
6	F	Anmatyerre	Alice Springs		Group	F2F
7	F	English	Alice Springs	Y	Group	F2F
8	F	Arrernte, Luritja	Remote community	Y	Individual	F2F
9	M	Arrernte, Luritja	Remote community	Y	Individual	F2F
10	F	Luritja	Remote community		Individual	F2F
11	F	Arrernte	Remote community		Individual	F2F
12	M	Luritja, Arrernte	Remote community	Y	Individual	F2F
13	M	Arrernte, Luritja	Remote community	Y	Individual	F2F
14	F	Luritja	Remote community		Individual	F2F
15	F	Pitjantjatjara	Remote community		Individual	F2F
16	F	Luritja	Remote community		Group	F2F
17	F	Luritja	Remote community		Group	F2F
18	F	Arrernte	Alice Springs	Y	Group	F2F
19	F	Arrernte	Alice Springs		Group	F2F
20	F	Arrernte	Alice Springs		Group	F2F
21	F	Arrernte	Alice Springs		Group	F2F
22	F	Arrernte	Alice Springs		Group	F2F
23	F	Arrernte	Alice Springs		Group	F2F
24	F	English	Alice Springs		Group	F2F
25	F	Arrernte	Alice Springs		Group	F2F
26	F	Arrernte	Alice Springs		Group	F2F
27	F	Arrernte	Alice Springs		Group	F2F
28	F	Arrernte	Alice Springs		Group	F2F
29	F	Warlpiri	Alice Springs		Individual	F2F
30	F	Luritja	Alice Springs	Y	Individual	F2F

**TABLE 2:** Profile of non-Aboriginal participants.

	Sex	Profession	Location	Duration of service in Central Australia	Remote experience (outside Alice Springs)*	Experience in women’s health	Interview medium and format**
1	F	PCP	Alice Springs	>5			Zoom
2	F	PCP	Alice Springs	<5			Zoom
3	M	PCP	Alice Springs	>5			Zoom
4	F	PCP	Remote	>10	Y		Zoom
5	M	PCP	Alice Springs	>5	Y		F2F
6	F	PCP	Alice Springs	>5		Y	Telephone
7	F	PCP	Remote	>5	Y		F2F
8	M	PCP	Remote	>10	Y		F2F
9	F	Specialist physician	Hospital	<5	Y		Telephone
10	M	Specialist physician	Hospital	>5	Y		Zoom
11	F	Specialist physician	Hospital	>10		Y	F2F
12	M	Specialist physician	Hospital	>5			F2F
13	M	Specialist physician	Hospital	>5	Y		F2F
14	F	Nurse	Alice Springs	>10		Y	Zoom
15	F	Nurse	Darwin/remote	<5			Zoom
16	F	Nurse	Remote	>10		Y	Telephone
17	F	Nurse	Alice Springs	> 10			F2F
18	F	Midwife	Hospital	>10		Y	F2F
19	F	Midwife	Hospital	>10		Y	F2F
20	F	Midwife	Alice Springs	>10		Y	F2F
21	F	Midwife	Hospital	>10		Y	F2F
22	F	Midwife	Hospital/AS	>10	Y	Y	F2F
23	F	Midwife	Remote/AS	>10	Y	Y	F2F
24	F	Midwife	Remote	<5	Y	Y	F2F
25	F	Social worker	Hospital	>10	Y	Y	F2F, group
26	F	Social worker	Hospital	>5	Y	Y	F2F, group
27	F	Community worker	Alice Springs	>10	Y	Y	F2F, group
28	F	Community worker	Alice Springs	>10	Y	Y	F2F. group
29	M	Community worker	Alice Springs	<5			F2F, group

*Current, previous or corresponding with present role. **Individual interview unless otherwise stated.

### Research Paradigm

A narrative approach guided the collection, interpretation and representation of participant contributions. Narrativity, often assumed to have its origins in Western ontological theories of persons as rhetorical beings (43), is predated by the ancient Indigenous practice of storytelling, or “yarning.” Yarning has been described by Aboriginal researchers as a fluid and revelatory oral engagement between Aboriginal peoples and their past, present and future, preserving cultural integrity while enabling Aboriginal people to positively reconstruct their lives (44). It is a practice that has been recognised in various qualitative studies in Aboriginal health (52–59).

As a research strategy, yarning held weight for this study, facilitating a familiar practice through which to discuss what for many Aboriginal participants was an unfamiliar disease entity, while promoting the emplacement of the HTLV-1 story where it belongs, with those very participants. Privileging Aboriginal participant contributions in the data analysis was paramount to this approach and pursued by the lead researcher (FF) as far as possible within the constraints of her non-Aboriginal status. Also integral, however, was the inclusion of non-Aboriginal voices in the dataset, a decision which functioned dually as a means of revealing the perceptions of a group without lived experience of HTLV-1, and an invitation to non-Aboriginal participants to explore possible reasons for HTLV-1 inaction (58, 60).

### Recruitment Strategy

Semi- and unstructured interviews were conducted over an 8-month period in 2020. Interviews ranged from 45 min to 1.5 h in length, totalling over 52 h of raw data which was manually transcribed by FF. The research question—to explore perceptions towards HTLV-1 in Central Australia—informed the aims of this endeavour, which were to examine understandings and experiences of HTLV-1 health contact, and, to interrogate the socio-cultural drivers for HTLV-1 transmission, including the interface between this virus and sexual and reproductive health.

Recruitment was progressed on a rolling basis via purposive sampling through existing connections, snowballing techniques and the establishment of collaborative relationships with health or community organisations (61, 62). Introductions formed an important part of the interview *pro forma*, initiating dialogue in a culturally safe way by beginning with a “social yarn” before progressing to a “research yarn” (63). All interviews commenced with a statement by each participant about their cultural background and motivations for facilitation or participation in the project. This functioned to reduce the researcher/researched binary and increase the opportunity for a more empowered participant experience (44, 64).

### Recruitment and Consent of Aboriginal Participants

All Aboriginal participants were recruited via a trusted health professional, colleague, community organisation or support person (65). Knowledge of one’s HTLV-1 status was not required for participation and consenting materials made it clear that participants were not being approached because they have HTLV-1. This approach was intended to maximise participation, serving as an acknowledgement of collective notions of wellbeing and health participation in Aboriginal communities (66), mitigating the risk of participant shame and stigmatisation, and promoting principles of safety, respect and confidentiality (67).

Consent was sought from Aboriginal contributors at various stages of participation (68, 69). Following introductions between the interviewing researcher and the participant/s, FF facilitated a discussion regarding the purpose of the study and de-identification and use of data. Participants then provided verbal and written consent agreeing for their contributions to be recorded using an audio recording device. The consent process was continued by “checking in” with participants during and after the interview, or during a subsequent visit to the community, organisation or workplace.

In the remote community, a poster about the study was hung at the community clinic, store and aged care centre during the periods when FF visited the community. One female remote community member responded directly to the poster, initiating her involvement in the study by attending the clinic and requesting participation. The remaining nine remote community participants were recruited via Aboriginal staff at the clinic, a non-Aboriginal remote primary care physician (PCP), and through Aboriginal and non-Aboriginal staff at the art and child-care centres. Ten of eleven community members who were approached for an interview accepted the invitation to participate. One remote community member who initially consented withdrew participation at the commencement of the interview due to the method of data collection using an audio recording device.

To facilitate the interview itself, a simple flipbook was developed at the Baker Heart and Diabetes Institute Central Australia using health education material from the “HTLV-1 community survey” (NHMRC 1088517) (19) and incorporating images from a video resource that was developed at the institute. The text for the flipbook was formed in consultation with a remote nurse, a remote PCP and four HTLV-1 researchers at the institute, two of whom were Aboriginal and had extensive experience in providing health education to Aboriginal people about HTLV-1 and other medical conditions. The project’s objective was to explore existing knowledges, not to develop health literacy tools or to provide clinical advice. The chosen remote community therefore was one where community members had previously participated in the HTLV-1 community survey (19) and who were more likely to have some knowledge of the virus. Participants who desired information beyond that included in the flipbook were encouraged to speak with their treating medical practitioner.

In the interest of preserving confidentiality, eight of the nine interviews in the remote community were carried out as one-on-one yarns in either the clinic or a private, outdoor space. In contrast, three of six interviews conducted in Alice Springs were carried out as collaborative yarns (63). The latter interviews took heed of the collective nature of the bodies represented; one yarning group bringing together female Aboriginal workers in health and the others female leaders in two Arrernte organisations. Cultural safety was ensured in the group interviews by the presence of a trusted person nominated by the participants who subsequently verified the interview transcript.

### Recruitment and Consent of Non-Aboriginal Participants

Non-Aboriginal participants were approached via email, phone call or in person at their workplace and provided with written information about the study including a letter of invitation and consent form. Fifteen of the interviews with non-Aboriginal participants were carried out face-to-face at the Baker Heart and Diabetes Institute Central Australia. The remaining interviews were conducted at participant’s workplaces, via video or telephone call. Two prospective non-Aboriginal participants declined participation. These prospective participants sought organisational approval from two Aboriginal community-controlled health organisations (ACCHOs) and this was declined (in one case due to involvement in the Federal Department of Health HTLV-1 longitudinal study; no reason for declination given by the second organisation). Three town-based Aboriginal-controlled community organisations (ACCOs) that provided health promotion programmes in Aboriginal communities also declined participation in the study due to increased servicing pressures associated with the SARS-Cov-2 pandemic and because HTLV-1 was not thought to be relevant to their work.

### Thematic Analysis

Conforming with recommendations in the decolonizing literature, a combination of deductive and inductive analyses were applied to the data, where an empowerment lens both instructed the dissection of narratives gathered and acted as a vehicle to see peripheral stories (70). The progression of themes mirrored the participants’ rhetorical passage through the interviews. These were:

1. An inconsistent clinical approach to HTLV-1,
2. Perceived challenges associated with sharing HTLV-1 knowledges,
3. The collective impact of HTLV-1, and collective responsibility for resolution,
4. Reflections on HTLV-1 and prolonged breastfeeding,
5. HTLV-1 as sexually transmissible infection, and
6. Imagined action.

### Ethics Statement

This study was carried out with the approval of the Central Australian Human Research Ethics Committee, reference number CA-19-3573.

## Results

### Participant Profile

A total of 30 Aboriginal participants were recruited to the study (Table 1). Twenty-eight Aboriginal participants represented Central Australian language groups (Southern and Western Arrernte, Anmatyerre, Luritja, Pitjantjatjara, and Warlpiri). Two other Aboriginal participants were from outside Central Australia. Nineteen Aboriginal participants were living in Alice Springs, ten were residents of a small very remote community and one was visiting Alice Springs from a second, very remote community. Three of the remote community participants were male. Twelve Aboriginal participants had experience as either an Aboriginal Liaison Officer (ALO), Aboriginal Health Worker (AHW)/Aboriginal Health Practitioner (AHP), or an Aboriginal Community Worker (ACW), the roles of which are to promote culturally safe healthcare services. With the exception of the participant who was visiting Alice Springs, all Aboriginal participants declined the offer of an interpreter and expressed a preference to be interviewed in English.

Three of 12 Aboriginal participants who had worked in health, and 8 of the total 30 Aboriginal participants had prior knowledges of HTLV-1. Two had provided cultural brokerage for the HTLV-1 community survey and two had participated in the survey (19), one had interacted with HTLV-1 as an AHP, one had read a media article about HTLV-1 posted on Facebook in 2018 and one was aware of, and disclosed, their own diagnosis of HTLV-1.

The non-Aboriginal sample (see Table 2) was largely comprised of healthcare professionals, which included 4 nurses, 7 midwives and 13 doctors; 8 PCPs and 5 specialist physicians based at ASH. Two further interviews were carried out as focus groups, one with two hospital-based social workers and the other with three community workers from a non-government organisation whose services included sexual health promotion. More than a third (10) of the non-Aboriginal participants were in managerial positions.

In Australia, remoteness is defined in terms of the road distance from services, with Alice Springs considered a “remote” location and surrounding communities “very remote” (71). The authors acknowledge that these definitions were developed to assist with the distribution of government services and are not necessarily meaningful for Aboriginal people (72). In the proceeding section, participants are identified with their place of employment or residence as follows; ASH (“hospital”), Alice Springs (“town”) or in a very remote community (“remote”). An asterisk is used to indicate Aboriginal participants who also work or have worked in health (e.g., female remote community member*).

### Theme 1. An Inconsistent Clinical Approach

Interviews with healthcare professionals revealed a spectrum of understandings about HTLV-1. Thirteen of 26 practitioners referred to their limited experience with the virus and 17 stated a need for guidelines to inform clinical practice. All healthcare professionals first learnt of HTLV-1 while working or preparing to work in Central Australia.

HTLV-1 knowledges were generally higher among hospital-based specialist physicians than other healthcare professionals based in Alice Springs. The knowledges of remote PCPs varied. Hospital-based specialists and PCPs trained at ASH were most likely to identify diseases associated with HTLV-1. Clinical manifestations including bronchiectasis, sepsis and strongyloidiasis, together with dermatological and neurological conditions were cited as examples of clinically recognised disease associations of HTLV-1. The ambiguity regarding whether or how an HTLV-1 agenda would progress was unsettling for hospital-based healthcare professionals, whose responses broadly established the perception that HTLV-1 is *probably underappreciated as a disease entity* and that *everyone [at ASH] feels uneasy about it (specialist physician 1).*

This was in contrast to the perceptions of physicians working in primary care in Alice Springs, for whom a consideration for HTLV-1 was largely absent from their practice. Four of the five town-based PCPs did not perceive HTLV-1 to be relevant to their work:

*It doesn’t change our clinical decision-making or anything like that. It’s just something we know is probably rumbling in the background to some degree. But um, we also know that overcrowding and poor diet and all that sort of thing are huge, or alcohol use, are huge contributors to disease as well.*

*(town-based PCP 1)*

Poor social determinants of health, the dominance of chronic, non-communicable diseases such as diabetes in the primary health setting, and the absence of HTLV-1 management protocols, seemed to undergird a perception among PCPs that HTLV-1 is not a clinically significant infection:

… *we have very high turnover (of staff). And people come in and someone gets a discharge summary that mentions it. And then there’s all this often, I think, an overreaction, of ‘oh they’ve got this terrible virus, I looked it up’. And [they] get quite distracted from the core business, which is about chronic diabetes and heart failure and kidney disease.*

*(remote PCP 1)*

*I wonder if [addressing] it (HTLV-1) would make that much of a difference in some ways. Because like you said, there’s no [antiviral] treatment for it. I wonder if it would just make people more anxious.*…*Like in the end, unfortunately our population are going to be dying before they get leukaemia [ATL].*

*(town-based PCP 2)*

The perception that a high burden of non-communicable diseases and a low life expectancy disqualified the inclusion of HTLV-1 in clinical practice consistently emerged in the data, although this was not the case for some remote PCPs:

*I guess there would be presentations that I wouldn’t have recalled being a potential effect of HTLV-1, and I would have a diagnostic dilemma. And then consider could this be HTLV? And then do the serology and it would be positive. And then I would refer on to Infectious Diseases and bit-by-bit really, cases were mounting up*… *you would think that it’s a significant problem, and because the two recognised conditions, the tropical spastic paraparesis [HAM] and the T-cell leukaemia [ATL] have both occurred in my small population. That seems well beyond coincidence.*

*(remote PCP 2)*

This participant raised three examples of residents with HTLV-1 associated inflammatory diseases (ATL, HAM and myositis) (6, 73, 74) in the remote community where they worked, but also articulated the tension that deterred most healthcare professionals in this study from engaging with HTLV-1: the dilemma of balancing patient self-management with the encumbrance of being unable to offer a clear treatment pathway:

*They’re relatively engaged with it (HTLV-1). They have been willing to get further tests. But that hasn’t necessarily helped them… When Aboriginal people are worried, they will take action. But then that’s hard when you don’t know what action they can take. I guess there’s enough understanding there [among Aboriginal patients] that there is this other infection that could be causing different diseases.*

*(remote PCP 2)*

One remote community participant disclosed his own positive HTLV-1 status to the interviewing researcher, reporting associated inflammatory symptoms which had led him to become more actively involved in managing his own health:

*This is something, sickness that’s in me that I know. You know, everybody can have that on the bloodline*… *When you’re older, that will come to you. So that’s why I always keep on coming in and checking out the clinic.*

*(male remote community member 1)*

### Theme 2. Sharing Knowledges With Aboriginal Patients

Notwithstanding the engagement with HTLV-1 by remote Aboriginal people, healthcare professionals were uncomfortable with the responsibility of sharing HTLV-1 knowledges with their patients. Attempts to convey HTLV-1 knowledges were a rarity for all healthcare professionals, and this was ascribed to the challenges of working in a two-world health setting:

*I wouldn’t do specific pre- or post-counselling like you’re recommended to do with HIV.*…*Because I don’t think the degree of um, health literacy is there for that to be beneficial to the people that we’re screening*.

*(specialist physician 2)*

*Even though I’m sure they’re [Aboriginal people are] able to understand this stuff um, there’s a*…*language problem, in having conversations about complexity much of the time. It’s not clear to me how much people want to know about HTLV-1. And so whether it’s right to start off those conversations, particularly when it’s not clear what the next steps should be with that information.*

*(specialist physician 3)*

These difficulties coincided with perceptions that discussing HTLV-1 with Aboriginal patients may be irrelevant to their preferences and could be unethical or detrimental to Aboriginal people. The imbalance of power between healthcare professionals and patients, whether conceded or not, was a noticeable dynamic:

*I try not to talk about it (HTLV-1) because I think we’ve got more important things to talk about.*…*Sometimes people come in and say oh, they told me I have this infection, what does this mean? So I have talked about it*…*I just really don’t know the burden of disease associated with it (HTLV-1). And we do know that there’s a high burden of disease associated with other things. It does seem appropriate not to use energy talking about that (HTLV-1) when there’s other things that need to be talked about.*

*(remote PCP 1)*

*And they (Aboriginal patients) often look to you for that decision-making as well because when you say to them, this is what you should be doing, not this. And they go, okay. And whether they do it or not is their choice.*

*(town-based midwife 1)*

#### Perceptions Toward Screening Practices

The finding that healthcare professionals avoid or experience difficulties explaining HTLV-1 to patients was further discussed by participants in relation to screening practices. The clinical impetus to test and the ethics of testing in the absence of treatment were central to this rationale. Hospital staff identified several situations in which patients were tested for HTLV-1: prior to commencing immunosuppressive therapy, in the presence of unexplained neurological signs and symptoms, as part of a diagnostic work-up for immunosuppression, in clinical situations in which ATL is suspected, prior to organ transplantation and as part of the blood-borne virus protocol in the renal dialysis unit. Nevertheless, physicians were reluctant to discuss the implications of a diagnosis with their patients:

*I can’t think of any specific patient where I’ve sat down and said, we’ve done this test, we’ve found out that um, that you are positive for this HTLV story (informal; story is a word used cross-culturally to refer to a body of ideas, beliefs or experiences).*

*(specialist physician 3)*

Perceptions that HTLV-1 has an ambiguous natural history and that the diseases associated with HTLV-1 are untreatable were cited as a fundamental impediment to testing or counselling patients by eight physicians (seven PCPs and one specialist physician) and one mid-wife. Among physicians, advancing medical knowledge was generally given priority over public health measures to reduce infection rates. Four of the five specialist physicians and five of the eight PCPs dwelt on the hierarchy of evidence regarding disease associations other than ATL and HAM, stating a need for greater causational certainty. Paradoxically, six of these physicians stated that they had not consulted existing literature regarding their views, though four had attended seminars about HTLV-1 at ASH where the clinical evidence for HTLV-1 associated diseases beyond ATL and HAM was presented. In contrast, non-medical professionals (nurses, midwives, social and community workers) and those who identified as Aboriginal were more likely to reiterate the need for public health measures to protect Aboriginal people from further transmission.

### Theme 3. Collective Impact and Responsibility

The effect of European colonization on Aboriginal Australians and specifically, the gap in Aboriginal health outcomes was seen as closely tied to inaction on HTLV-1 in Central Australia. Ten of 29 non-Aboriginal participants (two PCPs, two specialist physicians, two nurses, three midwives and both social workers) remarked that a virus affecting non-Aboriginal Australians which caused serious diseases and occurred with a prevalence similar to that of HTLV-1 in Central Australia would elicit an immediate response:

*Maybe that’s the thing that we all need to be thinking, is just to try and take the colour out of it and really, what would we seriously be doing you know, if this was happening in Sydney?*

*(remote PCP 2)*

The obligation for HTLV-1 action in Central Australia was seen to lie with those belonging to a higher social class who have greater capacity to instigate change; the scale of action required held not as an impossibility, but rather asserted as a human right:

*Are we all in this for our jobs? That’s the question that actually has to be asked.*…*It’s very easy to say that (HTLV-1) is not my responsibility, that’s somebody else’s responsibility*… *And Aboriginal people know that. [But] people have to want to see Aboriginal people improve for the right reasons with no self-gain in that*… *[to] do it because we’re human beings.*

*(female town-based Aboriginal participant 1)*

Although Aboriginal participants were made aware that most individuals do not develop currently recognised HTLV-1 associated diseases (3), they remained concerned that no attempt had been made to provide their communities with knowledge about this lifelong infection:

*I want to say for some of us, that could be late, you know. We might have already got infected, which is a shame*…*.[if] I find that I have it. You know, it’s just going to turn my world upside down. That’s when I’m going to worry about my partner and my kids, you know. What if they have it too? And my grandchild, you know?*

(male remote community member 2*)

*If people have known about this for as long as you say, the question more is, then what have all of those highly paid individuals been doing with that information and that knowledge, and what role have they played in withholding that information and ah, to some extent they’re culpable for Aboriginal people not getting relevant information and access to the services that they require*… *And if this (HTLV-1) is already occurring, then my guess is some of my people have died because of this.*

*(female town-based Aboriginal participant 1)*

In the remote community, HTLV-1 knowledges assumed a still more personal nature as participants reflected on the death of a community member due to ATL:

*I think I heard about this [time frame redacted] when somebody from this community passed away. Like, I think this is the story. ‘Cause they (the deceased) was happy and like, they didn’t know they had it.*…*And then it caught them later on.*

(female remote community member 1*)

Deaths resulting from ATL are uncommon, however, participant narratives revealed the profound psychological impact that such a loss can have on surviving residents of a small remote community (adult ERP, 140). Seven of ten remote community participants voiced concerns about HTLV-1 in the context of the death of this community member, including fears that their relatives, or they themselves might be infected. In contemplating HTLV-1, its silent transmission, possible progression to disease and its neglect by those responsible for public health, participants questioned their own and their family’s HTLV-1 status:

*Do we just have the same thing as them (the deceased)?*

(male remote community member 3*)

*Our people don’t even know about that, you know*…it’s [the virus is] like a ticking time bomb (male remote community member 2*).

Clearly, the HTLV-1 experience was very intimate to participants in this community.

Social risks that have the potential to overlap with experiences of HTLV-1 were conceptualised, and lamented, by many participants. One remote community member stressed the potential attribution of positive HTLV-1 status to promiscuity, and raised concerns that this might be associated with shame, and with social and family division:

…*So it’s like, really careful to talk to people about anything. Especially people in the same family*…*if family’s getting all cheeky (in this context, aggressive) or anything, then they’ll go and pull this out*…

(female remote community member 1*)

The same participant questioned the value of providing results of HTLV-1 tests to community members:

*What if you tell them they have this, and you have no medicine for it? How do you think they’re gonna feel? That’s what I’m thinking like, just don’t talk to them.*

(female remote community member 1*)

This view was not shared by others in the remote community. However, participants reinforced the requisite that the concept of HTLV-1 be demystified; that even in the absence of a cure, a clinical pathway, *the* ‘*what next’ (female town-based Aboriginal participant 1)*, be offered to affected individuals:

*If you give us the information, everyone talks about this. Education is power.*

*(female town-based Aboriginal participant 2)*

### Theme 4. Reflections on Breastfeeding

Consistent with the demands made by healthcare professionals working in the Aboriginal community-controlled health sector (21), HTLV-1 testing is not included in antenatal screening in Central Australia. Nevertheless, the HTLV-1 status of some women of reproductive age is known because they were tested for clinical reasons before pregnancy. Poor health literacy and uncertainty around securing informed consent was identified by healthcare professionals as a major obstacle to the instigation of antenatal testing:

*Rather than testing everyone, they only test a select group because they find to explain it (HTLV-1) and ensure, you know, informed consent, is too difficult. So that’s why it hasn’t happened.*

*(hospital midwife 1)*

Despite these challenges, however, participants who worked in maternal health also expressed their unease with the tension between equity in maternal care and the failure to include HTLV-1 testing in antenatal screening:

*You’re taking choice away from people. They can choose not to be tested, but they should have the right to have it offered.*… *I think it’s going to be a big issue here, in the long term. Once the full extent and it gets more publicised and more known. It’s going to be a serious problem for Central Australia. As in, a human rights violation*…*those children, if they become well-educated and stuff, you know, they may well have a case against the health system for not protecting their interests.*

*(remote midwife 1)*

In general, participants who worked in maternal health were unsympathetic to narratives in which the disclosure of HTLV-1 knowledges is resisted:

*What are they (healthcare professionals) frightened of? That people can’t wrap their heads around the fact that you know, you can be asymptomatic? Do they think that Aboriginal people can’t understand that concept?*

*(specialist physician 4)*

At the same time, the tension between sharing HTLV-1 information and the need to promote breastfeeding clearly preoccupied maternal healthcare professionals. Recognising the importance of breastfeeding to infants in remote Aboriginal communities, ASH guidelines recommend individualised management of HTLV-1 infected mothers according to her social circumstances and the family’s ability to provide safe alternatives to breast milk. Nevertheless, the acceptance of these guidelines remains low:

*We’re not going to do that. We’re not going to tell people at six months or twelve months, whenever the hell it says, to stop breastfeeding. If people are still breastfeeding at six months, the risks associated with not breastfeeding are huge*…*So I don’t think we liked that policy, and that’s where I got to.*

*(town-based midwife 1)*

*It’s like all guidelines, we do write them, but do I think my staff do it? No, but anyway, you have to do it to start.*

*(specialist physician 4)*

#### Breastfeeding Perceptions of Aboriginal Women

Aboriginal women reiterated the concerns that were raised by healthcare professionals, and which are incorporated in ASH guidelines, about difficulties in accessing safe alternatives to breast milk. The safety of breast milk substitutes was thought to be compromised by high cost, uncertain access, inadequate storage facilities, poor hygiene, substandard water quality in some remote communities, the high mobility of some families and social stigma. Breastfeeding was also identified with certain social benefits including having a soothing role in families under stress and contributing to reflective bonding between women who come together to feed. All participants were troubled by the ripple effect of interrupted breastfeeding on the interwoven experiences of attachment, food security and collectivity, particularly in a low-resource, remote setting:

*So then what? The bond between us and our children is impacted, the nourishment of our children is impacted*… *The [up to] $80 for formula, the $80 for the box of nappies. You’re already looking at $160 just to keep that little person surviving. And that’s $160 that doesn’t get to be spread around the household of 30 others, you know, with the overcrowding situation, and in line with what we do, with regards to our obligations [cultural, and to family].*

*(female town-based Aboriginal participant 1)*

Of the 12 Aboriginal women who shared their personal experiences, 10 reported breastfeeding well into the child’s second and third birth year. Breastfeeding cessation was often expressed as a response to the child’s strength and physical development as opposed to chronological intervals (75). Many women, including some who had bottle-fed their children, viewed breastfeeding as *best, natural*, and *right* for the baby. Breastfeeding was also identified as a protective factor aiding women to avoid peer pressures to drink alcohol or to use other recreational drugs. Midwives attested to this *positive health story when there are so many negative stories and statistics about Aboriginal health (hospital/town-based midwife 1)*. Some referred to the crucial and affirming intersection between breastfeeding and the right to nutrition, and to life (76).

Nevertheless, it was identified that young Aboriginal women may increasingly be choosing to bottle-feed. Several Aboriginal participants, especially older women, grieved this change. Others adopted a more flexible approach. There was a strong voice for greater inclusivity regarding bottle-feeding practices:

*But the people that work in the health, sorry, but white people, they think we’re breastfeeding really well when we’re sent home, you know? They (women) just do it here (at the hospital) just to satisfy the nurses and midwives.*…*They’ve already bought the bottles and everything on their way out. But nine times out of ten [statistic unverified] the person out of shame will tell the doctor what they want to hear.*

*(female town-based Aboriginal participant 3)*

…*we also have to be responsive to culture changes. So culture should evolve, and that does include about ways about how people bring their children up. So there’s changes in culture anyway that could impact on HTLV-1 and how it spreads. In this current generation, breastfeeding is being questioned.*…*They want different things to what their mums had. So they want a baby, there’s no question about that. And they love their babies very much. But they also still don’t want to be tied to that baby 24/7.*…*And they see bottles as more convenient for that. They see things on tele[vision], they see things on the internet.*…*They see other people bottle feeding and well, why can’t we do that? I don’t think anyone’s done actual research into who’s breastfeeding and who’s bottle, ‘cause that’s a touchy subject.*…*It’s such a colonial pressure to say the right thing to the right people.*

*(remote nurse 1)*

### Theme 5: Reflections on Sexual and Reproductive Health: HTLV-1 as a Sexually Transmissible Infection

Participants identified various structural deficiencies that impeded the promotion of sexual health in remote Australia. These included the absence of specific sexually transmissible infection (STI) education for community members, difficulties engaging with men due to long-standing challenges in employing male AHPs (female town-based Aboriginal participant 4*), and difficulties with cultural navigation in communities where there were no “Strong Women” (77) (remote midwife 1). Despite a desire to harmonise Western sexual health perspectives with Aboriginal realities, it was emphasised that entrenched power inequalities that impede on sexual health promotion remain unresolved:

*We’ve had lots of literature; we’ve had lots of research. We’re still researching it. We’ve had lots of education. The resources we’ve got to educate people about it, they change over the years for the generations. We are not getting anywhere with sexually transmitted infections. And a lot of it comes back to power and control*…*about women and saying no. And it’s really difficult, because you’re [as a health professional] an authoritative person with a lot of power. We have a medical model and an understanding in another perspective.*

*(remote nurse 1)*

Although many healthcare professionals had not categorised HTLV-1 as an STI, there was some agreement that *that’s where it fits (town-based/remote midwife 1)* and that *adding HTLV-1 to that umbrella is useful (specialist physician 5)*. Some participants regarded the incorporation of HTLV-1 into routine sexual health screening, counselling and contact tracing as overdue. Some participants, however, thought that including HTLV-1 as an STI may be misleading due to a perception in the community that STIs are treatable. Others referred to the possible low uptake of condom use in long-term partnerships (41).

In addressing these challenges, participants spoke of the influence that community mobilisation might have in reducing the sexual transmission of HTLV-1:

*If I think of the woman who has the gait problem, you know, her confidently knowing that it (HTLV-1) was the problem, even if nothing could be done about it. I think those people then might be able to start a conversation with their community to then look at people protecting themselves [with condoms].*

*(remote PCP 2)*

*Once a community knows about a certain thing, they definitely pass it on to the next lot you know, friends and family.*…*It might be a good idea to start in one community. Spread your wings from there.*

(male remote community member 3*)

*I mean, it’s about having a community that think it’s the place where we’re gonna crack this problem. To have people think that they really are important to the rest of the country, or at least the rest of the country they care most about.*…*With the possibility that it’s like yeast, it might actually make the dough rise.*

*(remote PCP 3)*

#### Comparison With HIV

Resonating with this notion of community initiative were numerous references to the mobilisation of Aboriginal communities during the HIV era (78–80). On learning that HTLV-1 was first discovered in Central Australia in 1988, Aboriginal participants were disappointed that attention to HTLV-1 was eclipsed by the dynamism of HIV initiatives:

*When I was in schooling there was a lot of talk about that AIDS thing but not realise that other disease (HTLV-1) there. But that other disease (HTLV-1) been here all those time.*

*(female town-based Aboriginal participant 6)*

*We knew about that (HIV). We knew it was coming. Difference with HTLV-1 is that already here. And we protected ourselves, you know? Using stuff that you know, when you [are] having sex and all that. To protect yourself. But this one is like a wolf in the night.*

(male remote community member 2*)

The parallels drawn between HTLV-1 and HIV by Aboriginal participants signalled that they conceived it possible to implement strategies to reduce HTLV-1 prevalence in their communities. The community workers and those healthcare professionals with international experience also emphasised the efficacy of HIV programmes and their potential application in Central Australia:

*I say that HTLV-1 sickness in the blood. They (Aboriginal people) understand me, you know. Blood cancer, sex things. It’s like HIV.*

(female town-based Aboriginal participant 5*)

### Theme 6: Imagined Action

The possibility of an HTLV-1 public health agenda was entertained by all participants; even those who were most doubtful about the virus’ clinical significance or the value of such an initiative. Participants were inclined to imagine HTLV-1 literacy developing on a continuum in which a consistent message is provided to individuals at various junctions of clinical care and occurring simultaneously with the development of community knowledges through health promotion; each component being equal in a multi-level public health endeavour (81).

Aboriginal participants expressed a desire to have HTLV-1 testing made available to them via culturally safe health messaging where community members are invited to request a test or discuss their known status. Epidemiological surveillance testing was not supported, and responses also indicated difficulties with obtaining consent for de-identified data linkage in research:

*Our mob, like, we just don’t want our stuff [blood] floating out there in never-never land. Don’t even know what’s touching with it because still, that’s part of us you know. That’s what I think, and I think most of us would agree, we just don’t want yous doing whatever.*

*(female town-based Aboriginal participant 2)*

Healthcare professionals and Aboriginal participants envisaged clinical and educational spaces where individuals could approach HTLV-1 knowledges at their own pace. A shared language, untroubled physical environment and the use of visual aids were enmeshed in the aspirations of both groups. Discussions about sexual wellbeing were identified as an important touchpoint for the promotion of knowledges:

*I think it’s safe to have those conversations. And they’re conversations that have to be had. It’s how we go about doing it.*…*I think we find now that a lot of health providers can break through having those conversations with the affected individuals.*

*(female town-based Aboriginal participant 1)*

Specific emphasis was placed on the complementary role of Aboriginal community members who work with health services. In particular, the readiness of Aboriginal people working in healthcare (ALOs, AHPs, AHWs, and ACWs) to provide cultural mediation—(re)presenting Western health paradigms and addressing colonial processes that repress the sharing of health knowledges between generations—was seen as a key attribute in navigating the sensitivities presented by HTLV-1 transmission. The resolve of participants who held this status testified to this position:

*I’d like to know more about this HTLV so I can explain it to my people.*

(female town-based Aboriginal participant 7*)

The suggestion of men’s and women’s wellbeing camps as a means to facilitate this knowledge transfer gestured towards a recognised practice in Aboriginal health (82, 83) that, according to older Aboriginal participants, dates back to the HIV era. Men and women shared previous experiences of going *out bush* specifically to talk about their sexual health. Integral to coming together was the stipulation that this occurred in gender-separate groups away from the community, ensuring cultural safety and confidentiality, and establishing the meeting as a protected yarning space (84). There was consensus that the transmission of HTLV-1 via sex and breastfeeding were appropriate discussion topics at such camps.

Despite the socio-economic challenges, Aboriginal women felt that the use of breast milk alternatives to protect their children from transmission was possible. Healthcare professionals, particularly those with a background in women’s health, were also supportive of action via community clinics that would assist remote women living with HTLV-1 to alter breastfeeding practises in order to reduce viral transmission. There was agreement that an integrated approach, where all healthcare professionals *own HTLV-1 (hospital physician 4)* would ensure that families are offered continuous support. However, Aboriginal women also highlighted the limitations of clinic-based, patient-centred care for infected women in families where parenting is non-nuclear (75), indicating that breastfeeding practices and how they relate to supporting women with HTLV-1 is an area for further exploration.

Finally, a chronic disease model was suggested as a framework for HTLV-1 patient management, with some healthcare professionals already incorporating principles of chronic disease management with HTLV-1 positive patients. Regular dermatological examination of a young child infected maternally from a mother with a high HTLV-1 PVL and end-stage bronchiectasis, was one example of this.

In the absence of direct acting antiviral agents and limited treatment options for HTLV-1 associated inflammatory diseases, the value of pursuing new HTLV-1 knowledges was recognised:

*Because we might be missing a whole lot of prevention and treatment. But we don’t know, so we need to be both researching and practicing. Bringing it (HTLV-1) online quickly and studying it at the same time, as opposed to a you know, a twenty-year study if you’re going to do it from birth. And that’s really too long.*

*(remote PCP 1)*

Importantly, the acknowledgement that an extant preventative approach may lead to improved community awareness of HTLV-1 challenged counter-narratives in the data, which postulated that HTLV-1 knowledges continue to be withheld from Aboriginal communities until the science and epidemiology of the virus is fully understood.

## Discussion

**FIGURE 2:**
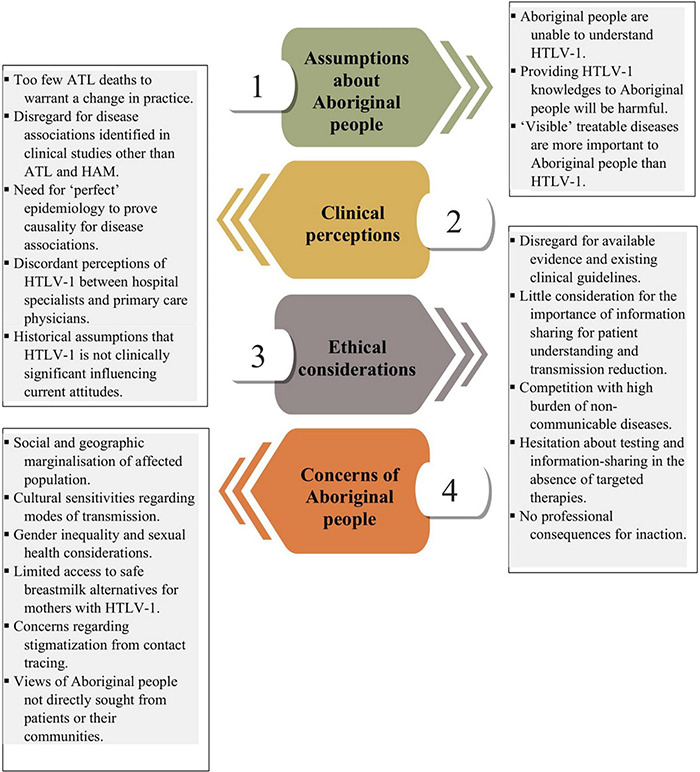
Barriers to the prevention of HTLV-1 in Central Australia. Modified figure © PresentationGO.

Although HTLV-1 was first found to affect Aboriginal Australians in Central Australia and the far north of Western Australia in 1988 (12), there has been no coordinated public health response to reduce transmission in this population. In the first qualitative study to examine perceptions toward HTLV-1 in Central Australia, we identify possible causes for this lack of progress across several thematic areas: (i) assumptions about Aboriginal people by non-Aboriginal health professionals; (ii) perceptions of HTLV-1 among these health professionals; (iii) ethical considerations, and (iv) concerns voiced by Aboriginal people themselves (Figure 2).

A salient barrier to HTLV-1 prevention in Australia is a focus on the low number of Aboriginal deaths resulting from ATL, a narrative historically rooted in the interpretation of Aboriginal socio-cultural beliefs by non-Aboriginal clinicians (85, 86) and the perception that HTLV-1 would not be considered a priority by Aboriginal people (87). Discordant perceptions of HTLV-1, particularly between hospital-based specialist physicians and physicians in primary care, continue to limit access to HTLV-1 healthcare and to obscure the representation of HTLV-1 to patients and their communities. While clinicians universally acknowledged that HTLV-1 has been a cause of death for some Aboriginal people, only 10 of 26 healthcare professionals in this study directly stated their support for a public health response to reduce the risk of HTLV-1 transmission among Aboriginal people.

Central Australia has the highest regional adult HTLV-1 prevalence worldwide (39%) (19), with a 50% prevalence among those older than 45 years (88). Each of the major clinically recognised HTLV-1 associated diseases have been reported in this region including HAM (7, 16), HAPD (19), uveitis, infective dermatitis (7, 89), and ATL (14, 15, 18), a condition that has proved rapidly fatal in this remote Aboriginal population. The evidence supporting a causal association between HTLV-1 and chronic lung disease has been recently reviewed (8), and bronchiectasis has been recognised as a disease association of HTLV-1 in international guidelines of the British Thoracic Society and the American Public Health Association (90, 91). Increasing HTLV-1 PVL has a strong dose response effect on the risk of developing ATL and HAM (92, 93), and this has been repeatedly demonstrated for HAPD in hospital (10, 11, 94) and community based studies in Central Australia (19, 88) where the risk of adverse clinical outcomes and death is substantially higher among those with a PVL exceeding one HTLV-1 copy per 100 peripheral blood leukocytes (19). Significantly, 36% of adults with HTLV-1 in a recent community survey had an HTLV-1 PVL exceeding this level (88). Similarly, HTLV-1 seropositivity is associated with increased mortality in all endemic areas where this has been studied (9).

The present study highlighted the impact of a single death due to ATL on surviving residents of an Aboriginal community who were unaware of their HTLV-1 status, provoking serious existential concerns with implications for individual and collective wellbeing. In Brazil, higher rates of depression and anxiety have been attributed to the risk of developing life-threatening complications of HTLV-1 (95). In the remote context of Central Australia, further social and cultural dislocation can result from complications of diseases such as HAPD and HAM, which require repeated visits to Alice Springs for treatment, and ultimate relocation to this regional centre if residence and associated care in very remote communities can no longer be supported (96).

Healthcare professionals in this study voiced concerns that Aboriginal people would find it difficult to grasp the complex nature of HTLV-1 and its associated diseases; a perspective that contrasted sharply with that of Aboriginal participants, who indicated that they wanted to increase their understanding of HTLV-1. Differences between PCPs and specialist physicians in their perceptions of HTLV-1 make the provision of clinically accurate information to Aboriginal people difficult, a situation rendered still more complex by the failure to fund HTLV-1 related public health literacy information independent from the activities of research institutes that have a potential conflict of interest in this area. Beliefs held by non-Aboriginal clinicians with regards to the capacity or desire of Aboriginal people to hold HTLV-1 information follow a historical trajectory in Central Australia that flows from similar views that were published 25 years ago (85) and which were more recently expressed by PCPs at the first Australian consultative HTLV-1 forum in 2018 (21). Notably, this Federal Department of Health sponsored forum was not attended by residents of affected Aboriginal communities (21). The resulting consensus statement did not support the implementation of a public health response to reduce viral transmission in the affected population (21, 97), a position that continues to deny productive pathways for HTLV-1 knowledge sharing in Central Australia.

The major outcome of the Australian consultative HTLV-1 forum was a longitudinal study (21), the purpose of which is to prospectively determine the impact of HTLV-1 on mortality and illness among Aboriginal people living in Central Australia (98). Whether Aboriginal communities must experience a specific level of morbidity due to HTLV-1 or a certain number of HTLV-1 attributable deaths before a public health response is implemented has not been made clear (98). However, the present study suggests that this approach has inadvertently encouraged Central Australian clinicians to disregard evidence of HTLV-1 pathogenicity while they await the results of what they perceive to be the definitive study. Significantly, due to the inability to exclude at baseline participants with pre-existing HTLV-1 associated conditions, such as neurological diseases not diagnostic of HAM (99) or small airway disease which is commonly associated with HAPD (8), such a study will not be able to accurately identify incident cases of most HTLV-1 associated diseases. Further, the reliance on data-linkage and medical records in this remote context (98) will substantially under-estimate true rates of these conditions (88) and it is extremely unlikely that the 5 year follow-up period (98) will be adequate to demonstrate an effect. The unrealistic expectations that have been raised around this study therefore pose further risks to Aboriginal people who may have to wait years for the results of a project with major limitations before they receive pertinent knowledge that could reduce the risk of HTLV-1 transmission in their communities.

Accordingly, and despite the evident risks posed by HTLV-1 infection in this highly endemic region, the majority of healthcare professionals interviewed avoided considering HTLV-1 associated diseases in their clinical practice, requiring that “causation” for entities other than ATL and HAM be established before they would either change their clinical practice or advocate for a public health initiative. Three of eight PCPs interviewed explicitly doubted the pathogenic potential of HTLV-1 despite acknowledging that ATL is a cause of death in Central Australia. The rationale for not supporting a public health response to HTLV-1 was based on the ethical principle of non-maleficence and the perception that sharing knowledges about HTLV-1 was likely to be harmful to Aboriginal people. For example, many healthcare professionals grappled with HTLV-1 testing as either “right” or “wrong” in an abstract moral sense that did not countenance any possible benefit to patients or their communities. Benefits to the individual that might be expected to result from securing a diagnosis, such as preventing futile cycles of investigation, accessing treatment for some HTLV-1 associated diseases, gaining access to specialist support and allowing patients to gain agency over the disease process were not considered by the majority of clinical participants, nor was the potential for any associated public health benefit acknowledged.

The failure to implement a strategy to reduce HTLV-1 transmission among Aboriginal Australians is in contrast with measures taken to prevent HTLV-1 entering the non-Indigenous Australian population. Since 1994, all Australian blood donors—who are drawn from a population in which HTLV-1 is not endemic—have been tested for HTLV-1 (100), and post-exposure prophylaxis with anti-retroviral drugs is provided without charge to clinical staff working in Central Australia who are occupationally exposed to HTLV-1 (101, 102). The Australian approach to its Aboriginal people differs markedly from that of two other developed countries in which HTLV-1 is endemic. In Canada, a single death due to ATL and fewer than 20 identified HTLV-1 cases among First Nations people in Nanavut prompted a rapid and comprehensive public health response (103–105). Similarly, HTLV-1 incidence rates fell by 80% in Japan (24) following the introduction of a systematic public health program that reduced infant exposure to HTLV-1 through antenatal screening and recommendations to limit the duration of breastfeeding (24). This was followed by a public policy response emphasising research, awareness-raising, care coordination and counselling by trained counsellors (106). In less wealthy countries, including Brazil and French Guiana, women with HTLV-1 have been advised not to breastfeed (38, 107). A more structured public health intervention appears likely in the North and Northeast regions of Brazil where infection rates are 1% and where there are long established, multidisciplinary HTLV-1 clinics providing care to those living with HTLV-1 (108–110). Similar clinics were established as part of the national retrovirology service in the United Kingdom, a country that is not considered to be endemic with HTLV-1 (111). Notwithstanding the extraordinarily high adult HTLV-1 prevalence in Central Australia (88), no funding has been provided for a similar dedicated HTLV-1 clinic in this region.

Although our data are drawn from a remote Australian context, many of the issues raised resonate with other affected populations. Establishing meaningful community-based control of HTLV-1 health literacy and clinical management is rendered difficult by the disenfranchised nature of communities impacted by HTLV-1 around the world (112). The stories of affected individuals are rarely in the spotlight of public health policy development, a marginality that is further exacerbated by the clinical consequences of HTLV-1 diseases that can limit social participation (42). In Australia and elsewhere, health professionals who have no personal experience of HTLV-1 and are largely derived from a privileged socioeconomic group dominate discussions about public health policy and determine what information is received by those who are directly affected. This is contrary to themes of empowerment, community control and agency that are emerging in approaches to sexual health promotion for other STIs in remote Australian settings (113–115) and which have been successful in reducing transmission and stigmatisation for HIV (80). A similar absence of HTLV-1 initiatives led participants in a study from French Guiana to express a preference for infection with HIV on the grounds that the virus was better understood by their communities (38).

Importantly, our study begins to draw attention to the sexual and reproductive challenges faced by remote Aboriginal women in navigating experiences of HTLV-1. Condom-less sex is considered to be the predominant pathway for HTLV-1 transmission globally (23) and in contrast to HIV, women are at much greater risk of acquiring HTLV-1 by sexual transmission than are men (116). Experiences of gender inequality therefore intersect with risk of infection among potential carriers (117, 118). Only two qualitative studies in Brazil have explored gender imbalances in relation to HTLV-1 (40, 41) and few Australian studies have highlighted the difficulties encountered by women negotiating condom use in remote settings (119, 120). Women with HTLV-1, who often reside in impoverished communities where adherence to recommendations to limit breastfeeding is extremely challenging (121), also bear most of the responsibility for making difficult decisions regarding breastfeeding (38, 39).

It was determined at the Australian consultative HTLV-1 forum in 2018 that offering families routine antenatal screening would place an unnecessary burden on Aboriginal women (21). Consequently HTLV-1 testing is not part of routine antenatal care in Central Australia (122) and advice to breastfeeding mothers with HTLV-1 who were previously tested for clinical reasons is individualised according to ASH guidelines (123). In the present study, Aboriginal women felt that the use of breast milk alternatives to protect their children from transmission was possible, a finding that resonated with a qualitative study with Noir-Marron women in French Guiana (38). The imagined support for mothers with HTLV-1 was a significant story in our data which, in the face of anticipated challenges, powerfully underpinned Aboriginal understandings of health, resilience and cultural continuity that transcend Western constructs of social determinants, risk and time (124–126). Supporting women with HTLV-1 and normalising their experiences by integrating HTLV-1 awareness into relevant aspects of women’s healthcare will be crucial to any attempt to prevent maternal transmission in Central Australia.

The present study was able to explore perceptions to HTLV-1 among representatives of three stakeholder groups: hospital-based specialists, primary care physicians and Aboriginal people residing in Central Australia. Narrative inquiry, or yarning, privileged the grounded contributions of research participants and in this study yielded a resonant metanarrative in which the perspectives of stakeholders could be compared, contrasted and reconciled. Nevertheless, several important limitations must be acknowledged. Although only one Aboriginal participant required an interpreter, the use of primary languages for other participants may have provided a richer data narrative, although it is notable that all other Aboriginal participants chose to discuss this difficult topic in English. A self-selection bias in which those more interested in the subject responded to calls for interviewees is also possible, in particular in a remote area where health professionals have a heavy caseload with many competing priorities. Attempts to involve three ACCOs were unsuccessful due to the demands made of their health promotion services by SARS-CoV-2, and two ACCHOs declined to participate (in one case due to involvement in the Federal Department of Health HTLV-1 longitudinal study). The study did not include the far north of Western Australia (the Kimberley region), where HTLV-1 has previously been identified (12). Further, our data relates to Central Australia and may not be relevant in other endemic HTLV-1 settings.

Nevertheless, the perceptions of healthcare workers in our study remained consistent with those in the published literature (21, 85–87) suggesting that their expressed opinions reflect those of a section of the healthcare workforce in this region. Local HTLV-1 clinical guidelines are being developed, the dissemination of which may alter the views expressed by some health professionals in this study. However, our study also found that the uptake of breastfeeding guidelines at ASH was compromised by the beliefs of staff members, and the provision of more comprehensive clinical guidelines is unlikely to change the perceptions of healthcare professionals unless these are accompanied by a suite of public health measures. Understanding the factors that influence the attitudes of healthcare professionals toward HTLV-1 in Central Australia will be critical to the successful introduction of clinical guidelines (Figure 2).

Capitalising on international interest in the high prevalence of HTLV-1 in Central Australia (127), in 2018 experts wrote an impassioned letter to the World Health Organisation (WHO) requesting that the organisation acknowledge the significance of HTLV-1 and support the implementation of prevention measures across all modes of transmission in accompaniment with the education of healthcare professionals and the general population (128). The recent addition of HTLV-1 to the WHO’s Department of Global HIV, Hepatitis and STI Programmes (129) may assist public health authorities to develop community-based strategies to reduce HTLV-1 transmission. Concerns about stigmatisation following contact tracing, however, were prominent in our study as they are in other settings (130), and this presents practical difficulties to the inclusion of HTLV-1 testing in the routine STI screening of marginalised populations. Nevertheless, a systematic public health response to prevent HTLV-1 infection is otherwise possible, and this is made still more important by the absence of effective antiviral therapies (131). Participants in the present study were able to foresee a structured prevention program in which strong, community-led health promotion is combined with a clear clinical pathway. However, the public health leadership and genuine engagement with Aboriginal communities that is required for such an approach is yet to emerge in remote Australia.

## Nomenclature

A discussion regarding the various appellations for Australia’s Indigenous populations was beyond the scope of this manuscript. We predominantly use the term “Aboriginal” as it broadly describes the cohort of populations in Central Australia. Conforming to international human rights language, on occasion we use the term “Indigenous” to locate the experience of Australian Aboriginal people within a global movement that recognises and prioritises Indigenous lives.

## Data Availability Statement

The datasets presented in this article are not readily available. Due to the sensitive nature of this research, participants of this study did not agreed for their data to be shared publicly and data additional to what is contained in the research article and supplementary material is not available. Requests to access the datasets should be directed to FF, ffowler2015@gmail.com.

## Ethics Statement

The studies involving human participants were reviewed and approved by the Central Australian Human Research Ethics Committee. The patients/participants provided their written informed consent to participate in this study. Written informed consent was obtained from the individual(s) for the publication of any potentially identifiable images or data included in this article.

## Author Contributions

FF and LE conceived the presented idea. FF developed the study’s theoretical framework and conducted the research and analysis, for which LE provided supervision. Both authors discussed the findings and contributed to the final manuscript.

## Conflict of Interest

The authors declare that the research was conducted in the absence of any commercial or financial relationships that could be construed as a potential conflict of interest.

## Publisher’s Note

All claims expressed in this article are solely those of the authors and do not necessarily represent those of their affiliated organizations, or those of the publisher, the editors and the reviewers. Any product that may be evaluated in this article, or claim that may be made by its manufacturer, is not guaranteed or endorsed by the publisher.
